# Integrated Hunting Strategies for African Swine Fever Control in Wild Boar: A Comparative Review of Experiences in European Continent

**DOI:** 10.3390/vetsci13040340

**Published:** 2026-03-31

**Authors:** Silvia Pavone, Clara Montagnin, Carmen Iscaro, David Ranucci, Francesco Feliziani

**Affiliations:** 1 National Reference Laboratory for Pestivirus and Asfivirus, Istituto Zooprofilattico Sperimentale dell’Umbria e delle Marche “Togo Rosati” (IZSUM), 06126 Perugia, Italy; c.montagnin@izsum.it (C.M.); c.iscaro@izsum.it (C.I.); f.feliziani@izsum.it (F.F.); 2 Veterinary Research Center on Wildlife, Department of Veterinary Medicine, University of Perugia, via San Costanzo 4, 06121 Perugia, Italy

**Keywords:** African Swine Fever Virus (ASFV), control activity, disease control, depopulation, epidemiology, hunting, risk factor, surveillance, wild boar

## Abstract

African swine fever (ASF) has become one of the most significant transboundary animal diseases affecting countries worldwide. Wild boars play a major role in virus persistence and in local spread through geographical contiguity, while long-distance and transboundary dissemination is more commonly associated with human-mediated activities, particularly the movement of contaminated pork products and materials. Hunting is frequently considered a central tool for disease control; however, its epidemiological impact remains debated. This review comparatively analyses the approaches adopted by countries of European continent affected by ASF, paying particular attention to the role of hunting within integrated management strategies. The review examines country-specific control measures, including surveillance systems, carcass search and removal, fencing, zoning, and population reduction policies. The analysis shows that successful eradication was associated with early detection, temporary suspension of hunting in infected core areas, rapid spatial containment through fencing, and intensive passive surveillance based on systematic carcass removal. Hunting was reintroduced only after containment, as a regulated depopulation tool under strict biosecurity supervision. In contrast, where the virus was already widespread at detection and containment measures were delayed or fragmented, intensified hunting alone did not prevent endemic persistence. These findings indicate that hunting is neither inherently protective nor detrimental; rather, its effectiveness depends on timing, coordination, and integration within a structured epidemiological framework. Effective control requires combining wildlife management with surveillance, biosecurity, and clear governance.

## Simple Summary

African swine fever (ASF) is a viral disease caused by the African swine fever virus (ASFV), which infects exclusively species belonging to the family *Suidae*. The disease is typically severe with nearly 100% lethality rate in domestic pigs and Eurasian wild boar (*Sus scrofa*), whereas other wild suid species remain asymptomatic and act as reservoirs of infection. Over the past decade, ASF has spread globally, causing major economic losses and posing a significant threat to the pig production sector. Because ASF virus can persist in the environment and wild boars can act as a long-term reservoir of the virus, controlling the disease is particularly challenging. Hunting is often proposed as a key tool to reduce wild boar numbers and limit the spread of infection, but its real effectiveness remains debated. This review explores how different countries of the European continent have used hunting within broader disease control strategies. By comparing countries that successfully eliminated the disease with those where it became endemic, we identified important differences in timing, coordination, and management. Successful cases were characterized by early detection, temporary suspension of hunting in infected areas, rapid installation of fencing, intensive search and removal of dead animals, and only later, carefully regulated population reduction. In contrast, where measures were delayed or poorly coordinated, hunting alone did not prevent continued spread. These findings indicate that hunting can support disease control only when it is part of a well-planned and coordinated strategy. This information may help authorities design more effective policies to manage future outbreaks.

## 1. Introduction

African Swine Fever (ASF) is a highly lethal viral disease affecting domestic pigs and wild boars caused by African swine fever virus (ASFV), recently reclassified as *Asfivirus haemorrhagiae* [1].

Although ASF is a non-zoonotic disease and poses no direct risk to human health, it is responsible for significant socioeconomic consequences in affected countries, disrupting livelihoods [2], threatening the conservation of certain wild species in some areas [3], and impacting the local food supply system [2]. ASFV exhibits an exceptional ability to persist in the environment, in carcasses, and in animal-derived products. It remains infectious for extended periods owing to its resistance to a broad range of pH values, temperatures, and autolytic processes, and can survive for several weeks within carcasses [4]. Low temperatures, including freezing conditions, further enhance the virus’s survival by preserving its viability [5].

Conversely, cooking (60 °C for 30 min) [6], exposure to extremely acidic or basic pH values (pH < 3.9 or >11.5 in serum-free medium) [7], and specific chemical compounds [4] can effectively inactivate it.

There are 24 (I–XXIV) genotypes of ASFV that have been described so far in Africa, and the ASFV genotype I is the first that escaped from Africa in 1957 [8,9]. Genotype II was first described in Madagascar and Mozambique but has now been detected in a further eight African countries including two West African countries [10]. In 2007, ASFV genotype II escaped from the African continent to Georgia [11,12], subsequently reaching numerous countries in Europe and beyond—including Asia, the Americas, and Oceania—and affecting both domestic pig holdings and wild boar populations [13,14,15].

To date, thirteen EU Member States have reported ASF in either domestic pigs or wild boar: Spain, Czech Republic, Estonia, and Hungary reported outbreaks exclusively in wild boar, whereas Bulgaria, Croatia, Germany, Greece, Italy, Latvia, Lithuania, Poland, Romania, and Slovakia reported outbreaks in both wild boar and domestic pigs. In European non-EU countries, ASF has been notified in the Animal Disease Information System (ADIS) in seven countries: Albania, Bosnia and Herzegovina, Moldova, Montenegro, North Macedonia, Serbia, and Ukraine [15]. Furthermore, ASFV has affected all countries in East and Southeast Asia, including China (2018), South Korea (2019), the Philippines (2019), Papua New Guinea (2019), and Timor-Leste (2020) [16,17,18]. In 2021, ASF genotype II reached Santo Domingo, spreading to both the Dominican Republic and Haiti [17].

The spread of ASF across global ecosystems has represented one of the most challenging epidemiological issues for veterinary services, wildlife managers, and the entire pig production sector for more than a decade [19,20,21].

ASFV is characterized by a complex epidemiological cycle. In general, three main transmission cycles are recognised for ASFV. The sylvatic cycle, which is particularly relevant in the African continent, involves the natural reservoirs of the virus—bush pigs, warthogs, and soft ticks (*Ornithodoros* spp.). Within this cycle, the virus circulates and is maintained and amplified without causing major clinical disease in the reservoir hosts. The domestic cycle encompasses all transmission routes among domestic pigs, regardless of the original source of viral introduction. This includes tick-mediated transmission (the tick–pig cycle) where *Ornithodoros* spp. are present, direct contact between pigs, contaminated fomites, infected feed or water, and, less frequently, routes such as artificial insemination with contaminated semen or mechanical transmission by insects. Finally, the wild boar–habitat cycle is characterised by viral circulation within Eurasian wild boar (*Sus scrofa*) population. In this cycle, the contaminated environment from secretions and excretions of infected animals, as well as from infected carcasses, plays a crucial role in sustaining infection in wild populations [22,23]. In addition, anthropogenic sources of contamination, such as improperly disposed food waste and pork-derived products, may provide further opportunities for virus transmission when accessible to wild boar or feral pigs, thereby contributing to both local persistence and potential reintroduction of the infection [24].

For these reasons, wild boar populations have become one of the main challenges to ASF eradication in Europe. High population densities increase the number of susceptible hosts, resulting in elevated infection and mortality rates and, consequently, in a greater accumulation of infected carcasses and environmental contamination. This self-reinforcing dynamic sustains viral persistence in the ecosystem and facilitates environmental transmission, potentially allowing the virus to spread into previously unaffected areas [19]. This remarkable environmental persistence, combined with the ecological complexity of the main wild host species—*Sus scrofa*—makes virus control not merely a matter of animal health management, but a broader challenge of integrated territorial and wildlife management [21]. Several modelling studies converge on conclusion that direct transmission between wild boar and carcass-mediated transmission are the core mechanisms sustaining long-term ASF endemicity. Gervasi and Guberti (2021) demonstrated that, in their spatially explicit, stochastic, individual-based model, virus persistence after ten years remained approximately 52% when only direct and carcass-mediated infection routes are considered; the addition of survivor-mediated transmission increased persistence only marginally, to about 57% [25]. Their findings strongly suggest that infected carcasses dominate infection dynamics, particularly during the endemic phase. Similarly, O’Neill et al., (2020) showed that environmental transmission, primarily via carcasses, is a key driver of ASF severity and long-term persistence, especially under conditions of slow carcass degradation [26]. These results are consistent with ecological observations where the remarkable stability of ASF virus in a variety of environmental matrices—including carcasses, soil, water, and meat—which enables efficient indirect transmission and contributes to environmental maintenance of the virus [27]. Conversely, studies adopting different modelling approaches that explicitly incorporate wild boar social structure, specifically the relationship between population density and boar contact rates, and the impact of heterogeneous contact networks, indicate that population density and social organisation can substantially influence ASFV transmission and the probability of ASFV endemisation in some specific context [28]. Thus, while there is substantial agreement that indirect transmission via carcasses is central to ASF endemic persistence, different models diverge on how much other mechanisms contribute and on how model assumptions (e.g., social structure, contact rates, etc.) can shift the perceived relative importance of these routes—with consequent implications on which intervention strategies (e.g., carcass removal vs. hunting) are predicted to be most effective.

The relationship between hunting practices and ASF control is complex and often controversial. While regulated and strategically planned hunting may contribute to population management and indirectly support surveillance efforts, uncontrolled or poorly coordinated hunting activities can facilitate viral spread by increasing wild boar movement, disturbing natural behaviors, and contaminated materials through human activity [19,29,30,31,32,33]. Human-mediated activities associated with hunting have been increasingly recognised as important drivers in the spread of ASF, not only through wildlife disturbance but also via inadequate biosecurity practices. In particular, the improper handling and movement of infected carcasses represent a major risk factor, as ASF virus can persist for long periods in carcass tissues and the surrounding environment, acting as a significant source of infection for both wild boar and domestic pigs [34,35]. The translocation of carcasses or carcass parts from the hunting site, as well as the disposal of contaminated leftovers, has been identified as a potential pathway for long-distance virus spread, especially when hunters transport wild boar products across regions or countries [36]. In addition, fomites such as hunting equipment, knives, clothing, and vehicles can become contaminated during hunting and carcass processing, acting as mechanical vectors for ASF virus spread [35]. Human-mediated transmission via contaminated materials is widely recognised as a key factor explaining both local persistence and long-distance jumps of the disease [36]. The movement patterns of hunters, including repeated access to different hunting areas and cross-border hunting activities, further amplify this risk by connecting otherwise epidemiologically separate wild boar populations [36]. Overall, insufficient application of biosecurity measures during hunting activities—including inadequate carcass management, unsafe field dressing practices, and poor decontamination of equipment and clothing—can significantly contribute to ASF spread, highlighting the critical need for targeted awareness and strict biosecurity protocols within the hunting community.

Conflicts between the hunting community and veterinary authorities are common and often stem from differing priorities: hunters generally aim to maintain sustainable populations and preserve recreational opportunities, whereas veterinary services prioritise disease control and strict biosecurity measures [19,37,38,39,40,41]. Therefore, the relationship between ASF and hunting lies at the centre of an intense and often polarised debate [29,38,39,42,43,44,45,46]. On the one hand, hunters are recognised as key stakeholders in reducing wild boar densities and in contributing to territorial monitoring. On the other hand, certain traditional hunting practices have been identified as potential risk factors for virus dissemination, particularly when not supported by adequate biosecurity measures [29,38,39,42]. This complex picture also encompasses the development of certified wild game supply chains, as well as the economic opportunities that stem from the management and control of the wild boar population [47,48]. This article aims to explore in detail the role of hunting in ASF control in affected countries, considering the relevant regulatory frameworks, scientific opinions, internationally recognised guidelines issued by major institutions, technical reports produced by expert groups, and reviewing the scientific literature available.

The objective is to provide a critical, non-ideological analysis of the relationship between wildlife management, hunting activities, and disease control, highlighting where and why hunting can serve as a useful tool, where it may pose risks, and which cultural and organisational changes are required for it to become fully effective within a modern wildlife management strategy. Finally, the article also considers the opportunities that appropriate hunting management could offer from both a public health and economic perspective by implementation of wild boar meat supply chain.

For clarity, the terminology used to describe wild boar management interventions is defined as follows. *Hunting* refers to general hunting activities carried out within the framework of recreational or regulated wildlife management, irrespective of the specific technique employed. The term *culling* is used in a broader sense to indicate the deliberate killing of wild boar through various methods (e.g., shooting at baiting sites, trapping, or other techniques), typically within wildlife management or disease control programs. *Depopulation*, instead, refers to coordinated and targeted interventions aimed at significantly reducing or locally eliminating wild boar populations as part of a structured disease eradication strategy. While these terms may overlap in practice, particularly where hunting activities are intensified for population control purposes, they are distinguished here to better reflect differences in objectives, scale, and regulatory framework across the analysed countries.

## 2. Materials and Methods

A comprehensive computerized search on the MEDLINE (via PubMed) was conducted to identify published peer-reviewed articles written in English, without a publication date limitation on the role of hunting in ASF control. The search was performed on 19 November 2025. The utilized blocks of key terms are available in Table 1. Key terms were combined using Boolean operators and MeSH terms specific for PubMed [49].

The collected data was recorded and managed using Excel (Microsoft Office 365). All references were screened by two independent reviewers (SP and CM) through title and abstract assessment, and studies irrelevant to the review’s scope were excluded. In addition to peer-reviewed scientific literature, this review also examined relevant regulatory frameworks, scientific opinions, and internationally recognised guidelines issued by major institutions, including the European Commission, the European Food Safety Authority (EFSA), the World Organisation for Animal Health (WOAH), and the Food and Agriculture Organization (FAO). Technical reports produced by expert groups and non-binding recommendations from professional associations (e.g., EUVET, GF-TADs, etc.) were also considered.

## 3. Results

### 3.1. Search Strategy

Overall, 92 articles were retrieved from the databases for the topic of hunting in ASF control (Table 1). After screening titles and abstracts—and, when necessary, the full text—65 articles were excluded because they were not relevant to the scope of this review. Ultimately, a total of 27 articles addressing the role of hunting in ASF control were included (Table S1).

### 3.2. Role of Hunting Activity in ASFV Control

Numerous approaches have been proposed to control ASF in wild boar populations, yet their effectiveness remains widely debated [50,51,52]. Differences in ecological contexts, wild boar population structures, hunting traditions, and institutional capacities have contributed to heterogeneous outcomes among countries. In this section, the various approaches adopted by countries currently facing, or that have previously faced, ASF are presented, with the aim of highlighting both the successes and failures of the implemented strategies, the time required to eradicate ASFV where eradication was achieved, and the main critical issues encountered. The analysis was conducted by first describing, on a country-by-country basis, the approaches adopted for ASF management, with a specific focus on hunting activities during epidemic and endemic scenarios; subsequently, the implemented measures were analysed within a comparative framework to facilitate the evaluation of different management options and to identify those that proved effective for ASF eradication.

#### 3.2.1. Czech Republic

Czech Republic successfully eradicated ASFV in all suids as of 19 April 2019, in compliance with Chapter 1.6. and Articles 15.1.2., point 2 of 15.1.3. and 15.1.31, of the OIE Terrestrial Code and in accordance with the information provided in WAHIS [53]. The national strategy combined rapid containment, strict biosecurity, intensive surveillance, and a highly regulated hunting system [54]. Following the detection of the first ASF-positive wild boars in June 2017, authorities promptly established an infected zone and surrounding buffer areas. Movement restrictions, reinforced biosecurity measures, and intensive surveillance were implemented immediately [46,55]. During the initial phase, all hunting activities within the infected area were suspended to avoid disturbing wild boar populations and inadvertently promoting virus spread [54]. Passive surveillance was a cornerstone of the early response: all wild boars found dead were required to be reported, collected, and subjected to laboratory testing. Systematic carcass searches were organized, and financial incentives encouraged both hunters and the public to report discoveries. This approach proved essential: of the 230 confirmed ASF cases, 212 (92%) were detected in carcasses, demonstrating the high epidemiological sensitivity of passive surveillance [53]. Once the infected area was stabilized and peripheral containment measures—such as electric fencing and odour repellents—were established, hunting was reintroduced under strict control, shifting the focus from containment to population reduction [53]. Key elements of this regulated hunting system included:

− Only individual hunting and trapping in buffer area around the fenced infected area was permitted; driven hunts were strictly prohibited to avoid dispersing animals.
− Participation was limited to selected, trained hunters who received mandatory instruction in biosecurity, carcass handling, and safe transport procedures.
− Each hunted animal was officially tagged, bagged, and transported to rendering plants for destruction and laboratory testing.
− Meat from the infected area was strictly prohibited from entering the food chain.
− Baiting was allowed solely to support selective hunting, whereas all supplementary feeding was forbidden [53].

Hunters were also actively involved in structured carcass searches, expanding territorial coverage beyond what veterinary services could achieve alone. This dual involvement—both in selective hunting and carcass detection—significantly strengthened the surveillance system. By combining strict movement controls, reinforced biosecurity in domestic pig holdings, systematic carcass detection and removal, and highly regulated hunting activities within the defined zones, the Czech Republic successfully contained the outbreak within a geographically limited area of approximately 89 km^2^ [53]. The last positive case was identified in April 2018, with no additional infections detected for over a year [53].

#### 3.2.2. Belgium

ASF was first detected in wild boar in Belgium in September 2018. The country promptly implemented a series of preventive and control measures and, after 26 months of coordinated management, regained its ASF-free status at the European Union (EU) level in November 2020. In December 2020, the World Organisation for Animal Health (WOAH) officially recognized Belgium as ‘all swine’ ASF-free [56].

Control of ASF in wild boar populations relied on a combination of restriction zones, passive and active surveillance, carcass removal, fencing, and targeted population reduction, implemented within a structured management framework. A provisional infected zone was established in September 2018, and all activities within this area were immediately suspended. This included a complete standstill of tourism, forestry activities, supplemental feeding of game species, and all forms of hunting, to avoid disturbing wild boar and potentially facilitating virus spread [56,57,58].

Enhanced passive surveillance was implemented as a second core measure, focusing on systematic searches and removal of wild boar carcasses. Hundreds of personnel were involved, including regional forestry services (77%), the military (11%), hunters (5%), other government departments (3%), sniffer dog teams (1%), and additional stakeholders (3%) [56]. Carcass searches were conducted in groups of six to eight individuals, supervised by local forestry officers, with an iterative schedule ensuring that adjacent areas were not surveyed on the same day. Search effort—quantified in terms of time spent and area covered—was systematically recorded and mapped. Hunters were required to report all carcasses, which were geolocated using GPS and reported via a mobile application to Civil Protection authorities for prompt removal [56,57].

A network of unburied wire fences, 1.2 m high, was installed along roadsides and existing landscape barriers to limit wild boar movements and slow viral dissemination. These fences increased landscape fragmentation, thereby enhancing containment efforts. Moreover, ASF-free enclosing buffer areas surrounding the infected zone, termed “white zones,” were established between the inner and outer fencing barriers [56].

Once the infected area was fenced and the white zones defined, hunting activity was reintroduced with a focus on organized population reduction. In the white zones, hunters were responsible for depopulation, primarily through driven hunts, while authorities supported culling using trapping and night shooting conducted by trained personnel. In the infected zones, where the wild boar population had already declined substantially due to natural ASF-induced mortality, hunting was reintroduced under strict control to further reduce the population. Here, culling was conducted exclusively by authorities, primarily through night shooting at baiting sites equipped with remote camera systems, complemented by driven hunting and trapping [56]. In parallel, active surveillance was implemented, with approximately 40% of hunted wild boars sampled and tested for ASF [58].

All culled wild boar in both infected and white zones were tagged, geolocated, and recorded, including information on date, location, sex, and age. Mortality data were systematically collected from the onset of the ASF outbreak, providing quantitative insights into reductions in wild boar density over time [56].

#### 3.2.3. Sweden

ASF was confirmed for the first time in Sweden in September 2023 following the detection of ASFV in wild boar [15,59,60]. This event marked Sweden’s entry into the ongoing global ASF epidemic that originated in Georgia in 2007 [59]. At the time of the outbreak, the Swedish wild boar population was estimated at approximately 300,000 animals, based on hunting statistics, traffic accident data, and Trichinella surveillance results [61]. Swedish authorities rapidly established a preliminary infected zone and implemented intensive control measures, primarily focused on movement restriction and carcass management in accordance with European Union legislation [61]. Following approximately one year of coordinated control and eradication measures, Sweden regained its ASF-free status in 2024 [15]. Control efforts focused predominantly on enhanced passive surveillance, including systematic search, geolocation, sampling, removal, and destruction of wild boar carcasses within the infected area [60,61].

Strict access restrictions were imposed within the infected zone, including a ban on entry into forests and rural areas, with exception of gardens, agricultural land, roads, and established sports facilities. These measures aimed to prevent indirect human-mediated spread of the virus via people, vehicles, and materials, while also minimizing disturbance of wild boar and limiting their movements beyond established home ranges [61].

Hunting activities were suspended within the restricted zones [15,61] and the infected area was progressively enclosed by fencing [62]. Existing barriers, such as major roads equipped with high game fencing (e.g., moose fences) and fenced railway lines, were incorporated into the containment system and accounted for approximately half of the perimeter [62]. The remaining sections were secured with robust fencing (BE-type), including wildlife passage tunnels where required, and extensions along major roads in the southern and eastern sectors to prevent wild boar movements, including swimming across water bodies [62]. Following the completion of fencing, wild boar depopulation measures were implemented both within the fenced restricted zone and in adjacent external areas [62]. These activities were conducted through close cooperation between the Swedish hunters’ organizations and the National Veterinary Institute. Specifically appointed and trained hunters carried out targeted culling using baiting sites equipped with camera systems, trapping, and night shooting with night-vision devices, which are permitted in Sweden. All culled wild boars were systematically sampled and destroyed [61,62]. To further limit wild boar displacement, existing baiting sites were maintained, and additional baiting points were established, while selected crop fields were deliberately left unharvested, with farmers compensated for the associated economic losses [62].

The European Commission officially recognized Sweden as ASF-free in late September 2024, confirming the effectiveness of an eradication strategy based on intensive control measures, systematic carcass searching by hunters, fencing of infected zones, and targeted culling [60].

#### 3.2.4. Germany

Despite the preventive border fencing installed along parts of the Polish–German frontier to reduce cross-border movements of infected wild boar, by September 2020 ASF had nevertheless become established in wild boar populations across three of Germany’s sixteen federal states—Saxony, Brandenburg, and Mecklenburg–Western Pomerania [39,63,64]. While the disease appears to have been successfully eliminated from wild boar in Mecklenburg–Western Pomerania, and control efforts have achieved substantial progress in large areas of Brandenburg, sporadic cases continue to be detected in both Brandenburg and Saxony. In addition, eight outbreaks have occurred in domestic pig farms across several federal states, including Brandenburg, Mecklenburg–Western Pomerania, Saxony, Lower Saxony, and Baden–Wuerttemberg [39,65]. Germany’s strategies to control and eradicate ASFV in wild boar were carried out within the framework of EU legislation and relied on a combination of complementary measures. First, an efficient passive surveillance system—reporting found dead or died through a road traffic accident (RTA)—was implemented to ensure early detection and monitor the epidemiological evolution of the disease. Moreover, systematic searching (active research), sampling, and removal of carcasses were conducted using a range of resources—drones, police helicopters, human search teams, and trained search dogs—to maximize the probability of detecting infected wild boar carcasses [66,67].

Active surveillance, namely the sampling of hunted wild boar, together with intensified hunting to decrease population density and reduce wild boar reproduction rates, represented an additional cornerstone of Germany’s approach [39]. Local hunters played a central role in implementing these measures. Their willingness to engage not only in intensified hunting, but also in carcass searches, sampling of dead wild boar, and compliance with enhanced biosecurity protocols was essential for the effectiveness of ASF surveillance and control [39]. Their detailed knowledge of local landscapes and wild boar dynamics provided a critical basis for designing interventions that were context-appropriate and operationally feasible. Furthermore, effective public outreach targeting multiple stakeholder groups—including seasonal agricultural and forestry workers and military personnel—was identified as fundamental to support coordinated action [67].

In Germany, the use of fencing and hunting to achieve wild boar population reduction did not follow a strictly linear sequence but rather evolved in multiple phases [68]. Following the initial confirmations in September 2020, hunting was banned within the inner zones, at least in the early phase of the epidemic, whereas in the outer areas hunting was rapidly intensified to reduce population density and support carcass detection across affected regions [16,66]. Between late 2020 and 2022, fencing interventions were substantially expanded and refined: temporary electric fences were upgraded to fixed wire barriers, and complex systems—including inner and outer rings as well as “white zones”—were established around infected core areas. In several cases, completion of fencing served as a prerequisite for launching intensified culling operations within the enclosed zones, demonstrating a strategic integration of physical containment and targeted depopulation [66,67,68].

More than €160 million was invested in fencing—both for white zones around infected areas and for the ASF protection corridor—and these structures proved highly effective in limiting the spread of infection through wild boar movements when constructed. Overcoming challenges in cooperation and information exchange with hunters ultimately enabled the reduction in wild boar densities to nearly zero within white zones between double fencing along border areas, supported by continued hunting in accessible zones, rigorous fence maintenance, and the establishment of dedicated carcass collection points [67].

#### 3.2.5. Italy

The Italian ASF epidemiological situation has been characterized by the coexistence of four distinct and partly overlapping scenarios, reflecting marked geographical, ecological, and socio-cultural heterogeneity.

Sardinia represents a unique and largely isolated context. The island has been affected by ASFV genotype I since 1978, most likely following the introduction of contaminated food waste. The persistence of the infection for several decades resulted from a complex interaction of geographical isolation, socio-cultural factors, and traditional farming systems, leading to endemic circulation of the virus [69]. In particular, the widespread presence of illegal free-ranging pigs—deeply embedded in long-standing agropastoral traditions—combined with insufficient biosecurity, was identified as a major driver of ASF persistence [69,70,71]. In this context, hunting played a marginal role in ASF control, and eradication efforts primarily focused on restructuring pig production systems and eliminating illegal pig populations. Throughout the period of ASF circulation, hunting was never prohibited, even in historically endemic areas; however, hunting activities were gradually subjected to increasingly strict regulations, which helped mitigate the risk of virus persistence and environmental spread. Ultimately, ASF eradication on the island was achieved through the implementation of the EFSA exit strategy targeting wild boar populations [72,73,74]. Sardinia was officially recognized as ASF-free following Commission Implementing Regulation (EU) 2024/2526 of 23 September 2024 [75], which lifted ASF-related restrictions for the region.

In contrast, mainland Italy has experienced a highly fragmented and evolving epidemiological scenario, one of them remains ongoing. The first confirmed ASF case in continental Italy outside Sardinia was reported on 7 January 2022 in Piedmont and was caused by ASFV genotype II [21,69]. Shortly thereafter, additional ASF-positive wild boar carcasses were detected in Liguria. In June 2023, ASFV was identified in wild boar in Lombardy, and by August 2023 several domestic pig outbreaks were confirmed in the same region [76]. In November 2023, AFV was also confirmed in wild boar in Emilia-Romagna [21]. More recently, in April 2025, ASF was detected in wild boar in Tuscany, resulting in the expansion of the north-western cluster to include five regions [77].

Parallel outbreaks occurred in central and southern Italy. On 5 May 2022, ASF was detected in a dying wild boar within a natural reserve in the northern metropolitan area of Rome (Lazio), followed by additional positive carcasses in the surrounding area. On 9 June 2022, the virus was confirmed in a semi-free-range pig farm located near the initial wild boar cases [76]. In May 2023, ASF was detected in wild boar in the province of Reggio Calabria and, shortly thereafter, in two semi-free-range pig farms. During the same period, ASFV was also identified in wild boar carcasses in Campania [76].

To date, the Lazio, Campania, and Calabria clusters have been successfully eradicated. Lazio was officially recognized as ASF-free in January 2025 by Commission Implementing Regulation (EU) 2025/164 [78], Calabria in October 2025 by Commission Implementing Regulation (EU) 2025/2189 [79], and Campania in November 2025 by Commission Implementing Regulation (EU) 2025/2388 [80]. Conversely, the north-western cluster remains active. In northern Italy, territorial characteristics—such as the presence of extensive forested and mountainous areas alternating with lowland plains—combined with delays and challenges in the implementation of key control measures, hindered eradication and contributed to a progressive expansion of the affected area [69].

Following the introduction of ASF in northern Italy, all measures required under national and international regulations were promptly adopted to prevent further spread [69]. Drawing on the successful Belgian experience, Italian authorities attempted to establish fencing around the initially infected areas in Piedmont and Liguria. However, administrative complexity significantly delayed fence construction, and the challenging morphology of the territory limited feasibility. As a result, by the time fencing was completed, ASF-positive wild boar had already been detected beyond the enclosed perimeter [69].

The regulation of hunting activities and wild boar depopulation in infected areas underwent a prolonged process of revision through a sequence of national ordinances issued since 2022, accompanied by specific derogations and amendments. During the early phase of the epidemic, hunting activities were largely restricted and replaced by selective culling measures, including night shooting from vehicles and the use of trapping cages in peripheral infected areas of restriction zone II (RZII) [81]. Within core infected zones, more incisive methods were permitted, such as *girata* hunts—a traditional Italian collective drive hunting technique involving no more than three tracking dogs and up to 15 participants [82]. Subsequent ordinances further tightened hunting regulations in RZII, ultimately prohibiting all forms of collective hunting for every animal species as well as banning wild boar hunting [83]. Later, depopulation strategies were introduced, re-authorizing driven hunting conducted with no more than three dogs per hunter or hunting group [84]. Meat from hunted wild boars could be authorized exclusively for regulated self-consumption or commercialization only after negative PCR results for ASF had been obtained. In the case of commercialization, the meat was required to be processed in an approved establishment and subjected to one of the risk-reduction treatments provided for in Annex VII of Commission Delegated Regulation (EU) 2020/687. In all cases, the meat could be distributed solely within the same restriction zone (RZII or RZIII) in which the animals had been harvested [85].

Despite these adaptations, control measures applied in RZII proved insufficient, and the north-western cluster expanded substantially, involving both wild boar and domestic pig populations [21]. Significant legal and operational changes were introduced in 2024. Following an EUVET mission in July 2024 [86], Italy submitted a shared roadmap to the European Commission outlining corrective actions to strengthen ASF control and eradication [15]. Viral Expansion Control Zones (Zone di Controllo dell’Espansione Virale, ZCEV) and more targeted wild boar population management were implemented [15,87]. The ZCEV was conceived as a flexible buffer area that may include Restriction Zones II and I as well as adjacent non-infected territories, with variable dimensions extending up to a maximum width of 10 km on each side, internally and externally, in relation to major road or motorway infrastructures and other physical barriers considered effective. Within these zones, intensive depopulation activities were implemented with the objective of creating a “white zone” free of wild boar, in combination with additional surveillance and biosecurity measures, in order to halt further ASF spread [88].

Outside ZCEV, restrictions varied by zone, but ASF testing of all hunted wild boar carcasses remained mandatory, with negative carcasses eligible for risk-mitigating treatments or self-consumption and positive carcasses mandatorily destroyed [88].

Despite encouraging outcomes in Lazio, Campania, and Calabria, ASF remains active in the north-western cluster, where new cases in wild boar continue to be reported by February 2026 [77].

#### 3.2.6. Latvia

ASF emerged in Latvia in June 2014, likely introduced from neighboring countries [41,89]. Initially, ASFV affected the eastern part of the country. In 2016, the virus spread abruptly to the central region and subsequently continued its progression toward western Latvia. This rapid shift, observed in 2016, may have been associated with human-related activities [90]. By October 2019, ASF had reached approximately 85% of the country [90]. Oļševskis et al. (2020) observed that, following an initial increase in ASFV prevalence, the incidence of newly infected animals declined over time in both eastern and western Latvia [90]. This decrease began slightly earlier in the eastern region, consistent with the earlier onset of the epidemic there. Simultaneously, an increase in seroprevalence was observed in hunted animals. The subsequent decline in seroprevalence in the easter region of Latvia by the end of the study period was considered a promising finding, suggesting possible onset of ASF elimination in wild boar population [90]. Overall, the wild boar population collapsed from approximately 74,000 individuals in 2013 to about 20,000 in 2019 due to the high lethality of the virus combined with intensified hunting pressure.

Latvia implemented strategies to counter ASF spread, primarily focusing on wild boar population management. The main measures implemented included population reduction, carcass notification and safe disposal, associated with a reenforced surveillance system, encompassing both active and passive surveillance [90]. Enhanced passive surveillance was never implemented due to the extensive size of infected territory [15]. Wild boar hunting was not restricted nationwide [15]. Campaigns and economic incentives aimed at increasing hunting pressure were introduced, some specifically targeting adult and sub-adult females to reduce reproductive potential and limit population growth. To enhance hunting efficiency, authorities authorized the use of previously restricted tools, such as sound moderators (silencers) and night vision devices [90]. Behavioral restrictions were implemented, including a ban on supplementary winter feeding, with baiting permitted exclusively for hunting purposes. Furthermore, to reduce the risk of infected animals’ dispersal, restrictions on driven hunts were imposed during specific periods [90]. Financial rewards were included for reporting carcasses and their safe disposal was organized by veterinary services or local municipalities [90]. Despite these efforts, the continued spread of ASF within the wild boar population in Latvia indicates that the implemented measures were insufficient to fully contain the epidemic [77].

#### 3.2.7. Hungary

ASF was first detected in Hungary in April 2018, with initial cases identified in wild boar populations. Since then, the infection has become established in several regions of the country [91], displaying an epidemiological pattern characterised by long-term persistence in wild suid reservoirs with temporal fluctuations in the number of detected cases [15,91,92]. In contrast to several other European Union (EU) Member States affected by ASF, Hungary has not reported any confirmed outbreaks in domestic pig holdings to date [15].

ASF control in Hungary has been implemented within the framework of EU Implementing Regulation (EU) 2023/594, through the application of national zoning and control measures proportionate to the assessed epidemiological risk and aligned with European Commission guidance on ASF prevention and eradication [15]. However, publicly available information on the practical implementation of several key intervention measures remains limited. In particular, no detailed data are available regarding the use of fencing as a tool to restrict wild boar movements in infected or high-risk areas. Similarly, information on the role of hunting and depopulation measures in ASF control is scarce. Available sources indicate that individual hunting of wild boar has been permitted in Restriction Zone I, and that depopulation measures have been applied at the national level [15]. Nevertheless, the specific hunting methods employed, the intensity of culling, and the coordination between hunting activities and surveillance objectives are not clearly described in the literature. Moreover, none of the three commonly adopted approaches to enhance active carcass research—namely the use of trained search dogs, unmanned aerial vehicles (drones), or organised human search teams—appear to have been systematically implemented in Hungary to increase the effectiveness of carcass detection and removal [15].

#### 3.2.8. Greece

The first confirmed outbreak of ASF genotype II in Greece was reported on 5 February 2020 in a small non-commercial pig holding. Clinical suspicion and subsequent laboratory confirmation of ASF virus infection in domestic pigs triggered the immediate implementation of emergency control measures, including the culling of all pigs on the affected holding, the establishment of protection and surveillance zones in surrounding administrative areas, and the reinforcement of biosecurity requirements and official inspections in pig farms [93]. In line with the European Union strategic framework for ASF control, Greece subsequently adopted Joint Ministerial Decision No. 147/21886/2021, which established a national programme for epizootic surveillance, prevention, and control of ASF in wild boar populations [92]. However, detailed information on hunting regulation and the specific methods applied for wild boar depopulation remains limited in the available literature. Existing reports indicate that wild boar hunting was permitted only in selected parts of the restricted areas, under the supervision of Regional Coordinating Bodies that monitored the activities of designated hunting groups. These measures contributed to a gradual reduction in wild boar populations at the regional level [92].

Between 1 September 2020 and 31 August 2021, no ASF cases were detected in wild boar through either passive or active surveillance, although the overall surveillance intensity and number of tested animals were relatively limited [92]. Following this initial incursion, Greece remained free from ASF in both domestic pigs and wild boar throughout 2021, as confirmed by retrospective surveillance data [92]. Nevertheless, ASF re-emerged in Greece during 2023–2024, with multiple outbreaks reported in small- and medium-sized commercial holdings as well as in backyard farms, alongside sporadic detections in wild boar [13]. As of December 2025, the disease remains present in the country, with new cases continuing to be reported in wild boar populations [77].

#### 3.2.9. Romania

Romania reported its first outbreak of ASF in July 2017, in a backyard pig holding located in Satu Mare County. The introduction of the virus was most likely linked to the extensive circulation of ASFV in both wild boar and domestic pig populations in neighboring countries, particularly Ukraine and the Republic of Moldova [94]. Since the onset of the epidemic, ASF has had a devastating impact on the Romanian pig sector, with more than 1.3 million domestic pigs affected. The first ASF case in wild boar was confirmed on 29 May 2018 [94], and from that time until the end of 2024 a total of 163,987 wild boars tested PCR-positive for ASFV [15]. All affected areas were placed under official surveillance by the National Sanitary Veterinary and Food Safety Authority (NSVFSA), in accordance with the control measures laid down in the European Commission diagnostic manual [95].

Concerning wild boar population management, hunting has been permitted using all legally allowed methods. However, biosecurity measures were implemented [15]. To support these activities, a “Guide to good hunting practices and biosecurity during hunting” was disseminated nationally through the NSVFSA to inform hunting ground managers, hunters and other stakeholders. Both passive and active carcass search strategies were implemented, with active searches carried out exclusively by trained personnel. Hunting ground managers were required to conduct regular patrols to detect wild boar carcasses and ensure their prompt removal; financial compensation equivalent to 12 L of fuel per 1000 ha per month was provided to support these patrol activities [15]. Moreover, active surveillance on hunted wild boars has been performed [15].

As, in Hungary, no data are available regarding the use of fencing to restrict wild boar movements in infected or high-risk areas.

The measures adopted were insufficient to contain the spread of ASF. The rapid evolution and extensive geographic spread of ASF clearly indicate a loss of effective control in both domestic pigs and wild boar populations. A major challenge in disease containment has been the inaccurate reporting of backyard pig holdings and wild boar population estimates, raising serious concerns about the overall effectiveness of the control strategy [94]. These weaknesses were also highlighted in the most recent DG SANTE audit conducted in 2021, which concluded that animal movements, biosecurity practices within and between backyard holdings, and animal registration remained largely uncontrolled. Similar deficiencies were identified in the management of ASF in wild boar populations [94].

During 2023–2024, a marked reduction in ASF cases in both domestic pigs and wild boars was observed, following the implementation of eradication measures by the NSVFSA, including molecular diagnostics, quarantine, movement restrictions, disinfection of facilities, vehicles and equipment, controlled feeding practices, and stamping-out of affected herds [95]. However, despite this improvement, Romania accounted for 91% of all EU restricted zones III in 2024, with its entire territory remaining under ASF-related restrictions [15] and new cases in wild boar and domestic pigs continue to be reported by January 2026 [77].

#### 3.2.10. Poland

Poland has been affected by ASF since February 2014, when the virus was first detected in wild boar on 17 February, followed by the first outbreaks in domestic pigs reported on 23 July and 8 August 2014 [96,97,98]. The most likely source of introduction was the cross-border movement of infected wild boars [96]. Since then, ASF has progressively spread within the wild boar population, with a steady increase in detected cases and a marked epidemiological shift observed in 2020, when a sharp rise in ASF-positive wild boars was recorded [98,99,100]. The severity of the epidemic showed pronounced spatial and temporal heterogeneity, with the highest impact observed in specific voivodeships, such as the peak registered in December 2018, when 210 ASF-positive wild boars were confirmed in a single region [96]. By 2024, Poland accounted for the highest number of ASF outbreaks in wild boar within the EU, representing approximately 30% of all notified cases, while 64.5% of the national territory was classified under ASF-restricted zones (I, II, and III) [15].

Since the early stages of the epidemic, both international and national legislation have been applied for ASF surveillance and control in Poland [101]. Passive surveillance of wild boars found dead or killed in road traffic accidents, together with active surveillance of hunted wild boars, has been systematically implemented [100]. In addition, carcass search activities have been carried out using different approaches, including trained personnel, detection dogs, and drones; however, these activities were not always implemented in a fully systematic or homogeneous manner across the territory [15].

In line with EFSA recommendations aimed at limiting ASF spread, radical reductions in wild boar populations were advised as early as 2015, extending up to 100–200 km from the last detected outbreak. Within core control areas, defined by a 50 km radius, systematic carcass removal was required, and wild boar density was targeted to be reduced to approximately 20% of the pre-outbreak population [102]. Hunting activities have been permitted within all restricted areas, including also “blue zones” (i.e., long-standing risk areas) with a preference for individual hunting, while depopulation measures were applied in areas adjacent to these zones [15]. Despite the implementation of intensive hunting measures, including within ASF-restricted zones II and III, and depopulation measures, the total number of ASF-positive wild boars continued to increase [103,104]. This trend has been justified with the high reproductive potential of wild boars which may have counterbalanced population reduction efforts [102].

As part of the response to ASF spread, physical barriers were also employed as a complementary control measure. In 2019, following the westward spread of ASF into the Lubusz province, solid fences were erected at a radial distance of approximately 10 km from detected outbreaks in an attempt to slow virus dissemination; however, this measure failed to prevent further spread [105]. This outcome contrasts with experiences in the Czech Republic and Belgium, where fencing contributed to successful containment of ASF within limited areas. In those cases, the virus was detected at a very early stage after introduction, when the number of outbreaks was still low, enabling rapid and effective implementation of control measures [16]. In Poland, by contrast, the detection of numerous outbreaks over large distances within a short time frame suggests prolonged and largely undetected virus circulation within the wild boar population prior to official identification [102].

Overall, analyses summarizing more than ten years of ASF circulation in Poland indicate that, despite extensive efforts by veterinary services, hunting associations, and administrative authorities, the disease continued to spread rapidly, affecting both domestic pig holdings and wild boar populations [77].

#### 3.2.11. Slovakia

Slovakia reported its first ASF outbreak in domestic pigs on 24 July 2019, followed shortly by the first confirmed case in wild boar on 8 August 2019 [15]. The emergence of ASF coincided with a period of historically high wild boar abundance, a condition that facilitated rapid spatial spread of the virus and resulted in high early prevalence, particularly in the eastern regions of the country [106]. By March–April 2021, ASF had spread extensively, with elevated detection rates reflecting intense virus circulation in wild boar populations [106]. In 2024, approximately 63% of the Slovak territory was classified under ASF restriction zones (I, II, and III), indicating widespread endemic circulation [15].

ASF surveillance and control in Slovakia are regulated through a combination of international and national legislation and are supported by updated methodological guidelines issued by the District Veterinary and Food Administrations. These guidelines address both hunting ground management and pig holdings and were developed in close collaboration with official veterinarians [107].

Passive surveillance represented the cornerstone of ASF detection in Slovakia and proved substantially more effective than active surveillance, in line with observations from other affected countries [106]. In 2024, PCR testing revealed a markedly higher ASF positivity rate in found-dead wild boars (31.4%) compared with road-killed (0.8%) and hunted animals (0.5%) [13]. Similar trends were observed in previous years [13], confirming that passive surveillance offers greater diagnostic sensitivity, as carcasses predominantly represent animals that died from ASF, whereas hunted wild boars largely originate from the apparently healthy fraction of the population [106]. Although carcass search activities were not fully systematic nationwide, organised searches were implemented within restricted zones [15]. These activities relied exclusively on trained personnel, often comprising hunters, forest workers, and hunting ground managers. Local hunters, benefiting from detailed knowledge of the terrain, were actively engaged following specific biosecurity training. Carcass searches were conducted during each visit to hunting grounds, averaging 16–24 h per area per week, with increased intensity during epizootic phases characterized by elevated mortality [15]

With regard to hunting activities and wild boar depopulation, Slovakia permitted hunting within restricted areas, adapting management strategies according to zoning. Individual hunting was allowed in restriction zone II, while both individual and collective hunting were permitted in restriction zone I and in buffer zones. Hunting pressure increased substantially during ASF-affected years, with a particular focus on adult animals, consistent with strategies aimed at reducing recruitment and limiting population recovery [15]. As a combined effect of ASF-related mortality and intensified harvesting, Slovakia experienced a pronounced decline in wild boar populations. These population declines were followed by a reduction in ASF incidence, particularly in eastern Slovakia, likely reflecting decreased contact rates and lower transmission potential within the remaining population [106].

Beyond passive surveillance, Slovakia put in place active surveillance in domestic pigs. Healthy pigs were systematically tested prior to movement and at slaughter throughout restricted zones, and random testing at farm level was implemented nationwide within these areas [15]. In parallel, active surveillance was applied to wild boars, with approximately 50% of hunted animals tested in restricted zones [15].

No information is available in the literature regarding the use of fencing as a measure to control ASF spread in Slovakia.

Despite these encouraging trends, ASF continues to advance westward toward Austria and Czech Republic, highlighting the need for sustained wild boar population management and enhanced cross-border coordination of control strategies [106].

#### 3.2.12. Estonian

ASF virus was first detected in Estonia in September 2014 in the wild boar population [38]. From the initial point of introduction, the infection progressed from east to west and, by the end of 2016, had affected almost the entire mainland territory, with the exception of the island of Hiiumaa [38,45].

The Estonian control strategy was primarily based on a substantial reduction in wild boar population density, combined with structured surveillance and systematic carcass management, alongside the reinforcement of biosecurity measures. These interventions were implemented with the active engagement of the hunting community, which played a central operational role in surveillance, carcass detection and removal, and population control activities [38,45]. To reduce wild boar density, wild boar hunting is permitted in the whole restricted zones [15] and selective hunting quotas were established by decree of the Environmental Board [108]. Specifically, hunting regulations required that at least 50% of harvested animals be females and 50% be sub-adults (6–12 months of age), with the objective of decreasing the reproductive potential of the population and limiting demographic recovery. Hunting pressure was further intensified through an increased number of driven hunts, the provision of financial incentives per harvested wild boar, and the controlled use of baiting practices, which were permitted exclusively under defined conditions within infected zones [38,45,108]. Given their extensive territorial coverage and detailed knowledge of local habitats, hunters were also entrusted with key responsibilities in passive surveillance and carcass management, both recognized as central components of ASF control in wildlife [38]. To minimize the risk of human-mediated spread of ASF virus, strict biosecurity protocols were enforced. All hunting equipment, including vehicles, clothing, and footwear, had to be thoroughly cleaned and disinfected after each hunting event. Vehicles used for transporting hunted wild boar or animal by-products were required to be leak-proof, and offal had to be collected in dedicated containers; disposal in the field was strictly prohibited [38]. Hunters were also required either to bury wild boar carcasses found dead on-site—at a minimum depth of 0.5 m, with prior disinfection of the surrounding area—or to transport them to designated incineration containers. Financial compensation was provided to support compliance with these measures (€70 for on-site burial and €42 for transport to an approved container) [38]. Notification to the regional office of the Veterinary and Food Board was mandatory for both hunted and found-dead wild boar. Hunters were also responsible for collecting appropriate biological samples (e.g., blood, long bone, spleen, or lymph nodes) and submitting them, together with epidemiological data—including age, sex, and precise geographic location—to the local Veterinary and Food Board (VFB) office. All harvested wild boar were required to be stored under refrigerated conditions and were not permitted to leave the hunting ground until laboratory test results for ASF were available. In the event of a positive result, storage facilities and all associated equipment had to undergo cleaning and disinfection under the supervision of an official veterinarian [38]. In some instances, following the detection of an ASF PCR-positive wild boar, targeted depopulation measures were implemented, requiring hunters to cull all wild boars within the affected area associated with the positive case [15].

Similarly to Slovakia, no information is available in the literature regarding the use of fencing as a control measure.

Nevertheless, despite the implementation of these measures, ASF continues to be reported in Estonia in both domestic pigs and wild boar, according to official notifications submitted to the World Organisation for Animal Health [77].

In the wild boar population, surveillance data—including samples from hunted animals, wild boars found dead, and road-killed individuals—indicate an increasing trend in ASF prevalence from 2020 to mid-2025, suggesting sustained and widespread viral circulation despite long-standing population reduction measures [77].

Similarly, in domestic pigs, ASF has continued to cause new outbreaks, resulting in substantial economic and production losses. Between June and September 2025, eleven outbreaks were confirmed, leading to the culling of more than 55,000 domestic pigs [109].

#### 3.2.13. Lithuania

Lithuania first reported ASF in January 2014, when two wild boars found dead tested positive by PCR [35]. Following this initial detection, the virus progressively spread throughout the country, becoming endemic in the wild boar population and posing a persistent risk to domestic pig holdings [90,91].

After the initial epidemic phase, Lithuania implemented a control strategy in wild boar primarily focused on population reduction [35,110,111]. Therefore, wild boar hunting is permitted in the whole country with no limitations [15].

Within the national ASF management framework coordinated by the State Food and Veterinary Service (SFVS) [112], hunters and hunting ground managers assumed a central operational role, acting as key stakeholders in surveillance, population management, and carcass control activities [110,112]. Hunting pressure has been intensified both temporally and spatially, particularly within designated restriction zones [110]. Individual hunting and the selective harvesting of adult and subadult females were promoted through financial incentives aimed at reducing the reproductive potential of the population [110].

In November 2015, financial incentives were introduced to support intensified and targeted hunting of female wild boar. Hunters received €50 for females aged 12–24 months and €100 for females older than 24 months. Between October and December 2017, incentives for females over 24 months of age were increased up to €300. In addition, year-round hunting of wild boar was permitted [110]. All hunted wild boar within infected zones were subject to mandatory sampling for ASF laboratory testing, and carcasses were required to be stored under refrigerated conditions until negative results were confirmed [112]. In ASF-free areas, a minimum annual number of samples per administrative unit was established to ensure continuous surveillance coverage. In the event of laboratory confirmation of ASF infection, the hunting area manager, under official veterinary supervision, was required to ensure the destruction of the infected carcass and any carcasses potentially exposed to cross-contamination (Order No. B1-265) [112].

In affected areas, hunters were also required to conduct systematic inspections of the hunting unit for at least one month following confirmation of ASF, with a minimum frequency of one inspection per week (Order No. B1-265) [15,112].

To strengthen passive surveillance, in early 2016 the Lithuanian government introduced a financial reward of €30 for reporting dead wild boar. This measure significantly increased carcass detection. Additional incentives for carcass reporting and burial in ASF-affected hunting grounds were introduced in September 2017 [110].

In 2025, 541 dead wild boars were sampled, with a PCR positivity rate of 78.2%, indicating continued high viral circulation [90].

No structured depopulation strategy explicitly designed for ASF eradication was implemented [15].

Similarly to Slovakia and Estonia, no information is available in the literature regarding the use of fencing as a control measure during the epidemic phase.

Despite the implementation of these measures, ASF continues to be reported in wild boar populations in Lithuania by January 2026 [77].

#### 3.2.14. Overview and Comparative Analysis of African Swine Fever (ASF) Control and Eradication Measures Implemented in Countries of European Continent

The main control and eradication measures implemented in the selected countries included in this review are summarized in a comparative framework in Table 2. The table provides an overview of the timing, epidemiological status, and key interventions adopted, including fencing, hunting management, depopulation strategies, and surveillance activities. This structured comparison aims to facilitate the interpretation of the measures applied in different epidemiological contexts and to highlight those approaches that were associated with successful ASF eradication. In addition, the table allows the identification of gaps and inconsistencies in the reporting of specific measures across countries.

A cross-country comparison of ASF control strategies highlights clear differences in the implementation, timing, and integration of key measures between countries that achieved eradication and those where the disease remains endemic.

Countries that successfully eradicated ASF (Czech Republic, Belgium, Sweden) shared a consistent set of coordinated interventions [15,37,53,54,55,57,61,62,91,92,105]. These included early detection at a limited spatial scale, rapid implementation of fencing to contain the infected area, temporary suspension of hunting during the epidemic phase, and intensive enhanced passive surveillance based on systematic carcass search and removal. In Belgium and Sweden, hunting was subsequently reintroduced in a highly regulated manner and primarily used as a targeted depopulation tool within restricted zones [15,55,58,61,91,105].

In contrast, countries with ongoing ASF circulation generally exhibited a different pattern. Fencing was often absent, partial, or implemented with delays, limiting its effectiveness in spatial containment in mainland Italy, Latvia, Hungary, Greece, Romania, Poland, Slovakia, Estonian, and Lithuania [15,37,55,66,69,90,91,92,93,95,96,98,99,103,105,107,109,110]. Hunting activities were frequently maintained or intensified across large areas without clear differentiation between recreational hunting and epidemiologically driven depopulation in Latvia, Hungary, Greece, Romania, Poland, Slovakia, Estonian, and Lithuania [15,37,52,55,83,85,87,89,91,92,93,95,96,98,99,104,105,107,109,110]. Enhanced passive surveillance was implemented inconsistently or at insufficient scale relative to the extent of the affected territory in Latvia, Hungary, Greece, Romania, Poland, Slovakia, Estonian, and Lithuania [15,37,55,90,91,92,93,96,99,104,105,107]. Detailed information on depopulation strategies was lacking in Latvia, Greece, Romania, and Slovakia, whereas information on carcass search intensity was missing in Greece, further complicating the evaluation of their effectiveness. Germany represents a notable exception, having banned hunting activities in restricted zones until an extensive fencing system was implemented. Subsequently, intensive and comprehensive enhanced passive surveillance and depopulation measures were introduced once the fencing was completed. However, despite these efforts, ASF incursion was not prevented, and eradication has not yet been achieved [64,66,67,68].

**Figure 1:**
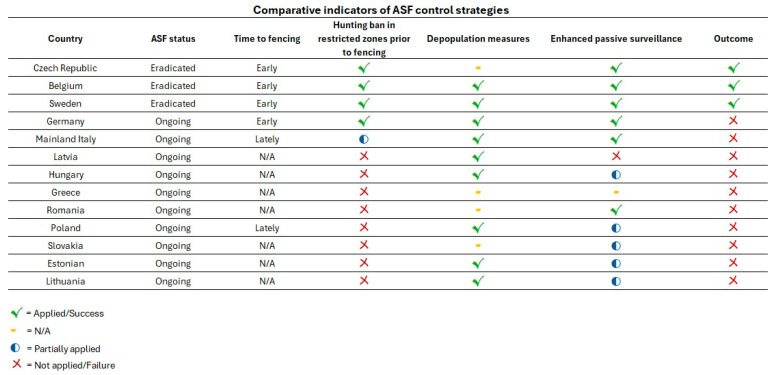
Comparative overview of the main control measures implemented across countries and their outcomes.

A comparative visual synthesis of the main control measures implemented across countries and outcome are presented in Figure 1.

## 4. Discussion

Human activity remains a major driver of both the persistence and spatial expansion of ASF in wild boar populations. While natural spread among wild boars is generally slow and spatially progressive, anthropogenic factors—including the movement of contaminated materials, inadequate biosecurity during hunting, carcass handling, and transport—can substantially accelerate viral dissemination [42,72]. In particular, non-compliance with biosecurity requirements during hunting activities has been repeatedly identified as a significant risk factor for both short- and long-distance spread, affecting wild boar populations and potentially bridging transmission to domestic pigs [19].

From an epidemiological perspective, optimal ASF control in wildlife relies on a combination of passive surveillance, structured wild boar population management, rapid carcass detection and removal, and spatial containment of infected areas [26,29]. However, the effectiveness of these measures depends not only on technical design but also on governance capacity, intersectoral coordination, and—critically—the willingness and motivation of hunters to actively participate under a veterinary-led framework.

The relationship between ASF and hunting therefore lies at the centre of a complex and often polarized debate. On the one hand, hunters are essential actors for territory monitoring, carcass detection, and population reduction. On the other hand, certain traditional practices—particularly driven hunting—have been considered potential amplifiers of virus spread [19,29]. The evidence reviewed demonstrates that wild boar population management through hunting and depopulation measures (hereafter referred to as wild boar removal) per se is neither inherently protective nor inherently detrimental; rather, its epidemiological impact depends on how, when, and under which regulatory framework is implemented. The comparative analysis presented in this review indicates that successful ASF eradication was not determined by the intensity of wild boar removal alone, but by the timing, coordination, and integration of wild boar removal within a broader, structured epidemiological strategy. Although in Slovakia reductions in wild boar population due to hunting pressure coincided with a decrease in ASF incidence, it is well established that the long-term persistence of ASF virus in infected carcasses can override expected density-dependent transmission patterns, allowing the virus to persist despite intensive population reduction and high mortality rates [23,104]. Countries that achieved eradication—such as the Czech Republic, Belgium, and Sweden—shared several operational features. First, ASF was detected relatively early, when the infected area was still geographically limited. Early detection, when combined with a prompt response, reduced the scale of intervention required and made spatial containment feasible. Second, hunting activities were immediately suspended within infected zones during the acute epidemic phase to prevent disturbance-induced dispersal of wild boar. During this phase, ASF-induced mortality often exceeds achievable culling efficiency. This dynamic can be strategically leveraged by temporarily suspending hunting activities, thereby reducing the risk of human-mediated long-distance spread and preventing the dispersal of infected animals beyond the core area. Third, rapid physical containment—primarily through fencing—was implemented to delimit the infected zone. Fencing did not act as a standalone solution but functioned as a spatial stabilizer, enabling subsequent targeted depopulation under controlled conditions. Fourth, substantial resources were invested in enhanced passive surveillance. Systematic carcass searching, geolocation, safe removal, and laboratory confirmation formed the backbone of early virus elimination. In all eradicated contexts, the majority of ASF detections derived from found-dead wild boars, confirming the superior diagnostic sensitivity of passive surveillance compared with testing of hunted animals, which predominantly represent clinically healthy individuals [15]. This observation is consistent with modelling studies identifying carcass-mediated transmission as a key driver of long-term persistence [25,26,28].

In contrast, countries where ASF became endemic or spread extensively exhibited a different epidemiological trajectory. In several Eastern European settings, the virus was already widely distributed at the time of official detection, limiting the feasibility of spatial containment [15,37,45,55,66,89,90,91,92,95,96,102,103,104,105,107,110]. Fencing was absent, partial, or implemented too late to effectively restrict movements. Although passive surveillance was formally established, systematic and scalable carcass search activities were often insufficient relative to the size of affected territories [89,98,99,102,103,104,106,107,109,110,113]. Hunting generally continued within restricted zones and was sometimes intensified; however, it was not always embedded within a clearly phased strategy distinguishing recreational activity from structured depopulation aligned with epidemiological objectives [52,83,85,87,88,89,90,93,95,96,98,99,103,106,107,109,110,114]. Even where marked reductions in wild boar density were achieved—such as in Latvia or Slovakia—the virus persisted, suggesting that population collapse alone is insufficient when environmental contamination and carcass-mediated transmission remain widespread. These outcomes must also be interpreted in light of external epidemiological pressures. Some affected countries are characterised by extensive and permeable borders with ASF-affected regions where wild boar populations move freely, contributing to continuous reintroduction risk and complicating the evaluation of national control programmes [115,116]. The north-western Italian cluster represents a intermediate and instructive case. Here, delayed implementation of fencing, and frequent regulatory revisions affecting hunting activities in both restricted and ASF-free areas generated operational instability [69]. Repeated amendments and derogations—partly aimed at maintaining socio-economic and recreational activities, including hunting—may have contributed to continued virus circulation and territorial expansion [21,69,83,85,87,88], illustrating how delayed containment and governance fragmentation can compromise otherwise robust technical measures.

**Figure 2:**
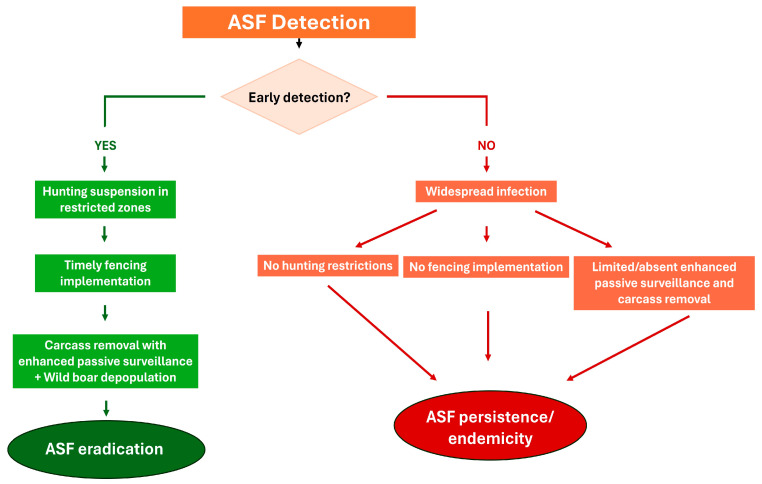
Conceptual framework of African swine fever (ASF) control pathways in wild boar populations. The flowchart illustrates the key divergence in epidemiological outcomes depending on the timing of ASF detection and the subsequent implementation of control measures.

Across all scenarios analysed, one consistent finding emerges: early detection, combined with the timely and temporary suspension of hunting activities and the rapid implementation of fencing around infected areas, appears epidemiologically central to achieving ASF eradication (Figure 2). Conversely, in countries where hunting was not suspended but instead promptly intensified across the entire territory without spatial limitation, and where fencing was not implemented—even when biosecurity measures were formally applied, as observed in Lithuania and Estonia—eradication was not achieved. Moreover, in settings where territorial scale, logistical constraints, or limited institutional coordination hindered systematic carcass detection and removal, the effectiveness of hunting as a control tool was markedly reduced. These observations further support the concept that hunting alone, even when intensified, cannot compensate for the absence of spatial containment and structured passive surveillance.

A further relevant finding emerging from this review concerns the substantial heterogeneity and, in some cases, incompleteness of publicly available data on ASF control measures. As summarized in Table 2, key operational details—such as depopulation methods, spatial extent and technical characteristics of fencing, and the systematic organization of enhanced passive surveillance—were often insufficiently documented, particularly outside the EU where reporting systems are not embedded within a harmonised supranational regulatory framework. Within the European Union, ASF management is supported by a structured legislative and governance system, including Implementing Regulation (EU) 2023/594 [81] and related technical guidance, which provide recommendations for surveillance, zoning, hunting regulation, and biosecurity measures in affected areas [117]. The EU framework also relies on a standardized flow of information from Member States to the European Commission, complemented by scientific assessment by EFSA, thereby promoting coordinated surveillance and control strategies across the Union. Although differences in implementation persist, this structure enhances transparency and comparability of national measures. In contrast, in settings where such harmonised reporting mechanisms are less formalised, the scarcity of detailed operational information reduces the ability to critically evaluate which specific components of national strategies contributed to successful containment or, conversely, to persistent virus circulation. This gap limits the development of robust, evidence-based best practices. Addressing these shortcomings would require greater international data sharing, standardized reporting of operational indicators (e.g., such as hunting intensity, hunting methods and associated restrictions, carcass search effort and applied techniques, and fencing specifications), and improved transparency in documenting field-level interventions. Given the transboundary nature of ASF and the ecological continuity of wild boar populations across national borders, strengthened interdisciplinary collaboration—integrating veterinary, ecological, epidemiological, and wildlife management data—appears essential. Enhancing these aspects would not only improve scientific understanding of ASF dynamics but also support more informed policy-making and adaptive management in future outbreaks.

A further limitation of this review relates to the nature of the data sources analysed. The study primarily relies on publicly available information, including scientific publications, official reports, and regulatory framework. While this approach ensures a consistent and comparable analytical framework across countries, it does not fully capture the socio-behavioural and operational dimensions of ASF control at field level. In particular, the perspectives, motivations, and constraints of key stakeholders—such as hunters, veterinarians, and wildlife managers—remain only partially represented in the available literature. These human and institutional factors, however, play a critical role in the implementation and effectiveness of control measures, especially in relation to compliance with biosecurity protocols, participation in surveillance activities, and acceptance of restrictive policies. Future research would benefit from integrating qualitative and participatory approaches, including interviews, surveys, and stakeholder-based studies, in order to better understand the practical challenges and decision-making processes underlying ASF management in different epidemiological and socio-cultural contexts.

Finally, beyond their epidemiological role in ASF management, wild boar population control measures may also generate opportunities from both public health and economic perspectives through the development of structured wild boar meat supply chains. Traditionally, wild game meat was marketed locally with limited regulatory oversight, resulting in variability in hygiene standards and product quality. However, recent regulatory developments within the European Union—particularly Regulation (EC) No 853/2004 [118]—have significantly strengthened the framework governing the commercialization of wild game meat, introducing mandatory training requirements for hunters, traceability obligations, approved game-handling establishments, and veterinary inspections at critical points along the supply chain [119]. Within this structured system, hunters are recognized as primary producers, responsible for appropriate harvesting, initial carcass inspection, hygienic handling in the field, and documentation of traceability data. In this context, wild boar meat represents a potentially sustainable, high-value protein source within an increasingly formalized game meat sector [120,121]. The integration of wildlife population management with regulated meat production aligns ecological objectives with economic valorization, consistent with circular economy principles. Moreover, regulated harvesting can contribute to public health by ensuring systematic inspection for zoonotic agents and improving traceability, thereby reducing the risks historically associated with informal marketing channels [122]. From an epidemiological and ASF control perspective, hunting activities should be integrated within a coordinated management framework that includes also ASF testing of all hunted wild boars when hunting is permitted within restricted zones. In some countries, such as Italy, regulatory provisions allowed the regulated commercialization or self-consumption of wild boar meat only after negative PCR results have been obtained [85]. However, this approach should be considered with caution, as it entails a non-negligible residual risk, particularly if traceability systems, laboratory turnaround times, and biosecurity procedures are not rigorously enforced. Conversely, hunting in ASF-free areas may contribute to maintaining wild boar densities within ecologically sustainable levels while also supporting meat valorization and stakeholder engagement. The management of hunted carcasses should therefore occur within a regulated and fully traceable supply chain, accessible exclusively to animals harvested in ASF-free areas or, in the case of restricted zones, only after laboratory testing has definitively excluded the presence of ASF virus. Such a structured supply chain would pursue a dual objective: enhancing the economic value of harvested animals and, more importantly, ensuring complete traceability and sanitary oversight at all stages, so that any ASF-positive animal is managed under strict biosecurity conditions and disposed of in accordance with veterinary legislation. Overall, prioritizing regulated wild boar meat production within structured game meat systems may represent a synergistic approach, linking population management, infectious disease control, food safety, and rural economic development. Continued investment in hunter training, standardized hygienic procedures, epidemiological monitoring, and transparent traceability systems will be essential to support the sustainable expansion of wild boar meat supply chains at both European and global levels.

## 5. Conclusions

This review demonstrates that hunting can contribute meaningfully to ASF control only when redefined from a traditional wildlife management or recreational activity into a structured veterinary intervention embedded within an integrated disease-control strategy. Its effectiveness depends not on its intensity alone, but on its timing, effective coordination between veterinary authorities and hunting communities, and alignment with epidemiological objectives. In the absence of these conditions, intensified hunting alone does not prevent virus persistence and may, under certain circumstances, increase disturbance and spatial spread. Therefore, the debate should not focus on whether hunting is beneficial or detrimental in absolute terms, but rather on how, when, and under which governance framework it is implemented.

The experiences reviewed here demonstrate that successful ASF eradication requires not only technical measures but also cultural, organisational, and institutional adaptation within the hunting sector—transforming hunters from primarily autonomous stakeholders into fully integrated operational partners within structured wildlife disease management systems under the coordination and governance of veterinary authorities.

Successful eradication experiences showed that only after ASF containment had been secured hunting can be reintroduced in a structured and highly regulated manner, primarily as a targeted depopulation tool within fenced areas or buffer zones and under strict biosecurity supervision. In these cases, hunting must not be treated as routine wildlife management but as a veterinary intervention embedded in a hierarchical control strategy: containment first, population reduction second, always accompanied by reinforced surveillance and carcass management. Moreover, the success of ASF control strategies depends on the active involvement of hunters as operational partners within regulated frameworks, supported by training, incentives, and institutional coordination. In contrast, where these conditions were not met—due to delayed detection, large-scale spread, limited coordination, or continuous external infection pressure—hunting alone proved insufficient to prevent virus persistence.

Finally, improving the availability and standardisation of data on ASF control measures, together with strengthened international collaboration and interdisciplinary integration, will be essential to support evidence-based decision-making and adaptive management of this challenging disease.

## Figures and Tables

**Table 1:** Results of the search strategy adopted to investigate the role of hunting in ASF control.

	PubMed: Original Search on 19 November 2025	Results
#1	African Swine Fever Virus OR ASFV OR African Swine Fever OR ASF	6429
#2	wild boar OR wild boars	29,257
#3	hunting OR hunting activity	12,560
#4	control OR surveillance	8,911,449
#5	#1 AND #2 AND #3 AND #4	92

**Table 2:** Overview of African swine fever (ASF) control and eradication measures implemented in the European continent.

Country	Start Date	ASF Epidemiological Status	Fencing	Hunting Activity	Depopulation Measures	Passive Surveillance	Enhanced Passive Surveillance	Active Surveillance on Hunted Wild Boar
Czech Republic	June 2017	Eradicated (April 2019)	Yes	Initially banned nationwide; after fencing, only individual hunting and trapping allowed in buffer zones	N/A	Yes	Yes	No
Belgium	September 2018	Eradicated (November 2020)	Yes	Initially banned nationwide; after fencing, hunting shifted towards depopulation activities	Yes; night shooting at baiting sites with remote cameras, driven hunting and trapping	Yes	Yes	Yes
Sweden	September 2023	Eradicated (September 2024)	Yes	Initially banned in restricted zones; hunting shifted towards depopulation activities	Yes; depopulation implemented within fenced zones and adjacent areas	Yes	Yes	Yes
Germany	September 2020	Ongoing	Yes	Initially banned in restricted zones and reinforced in the outer	Yes; after fencing, depopulation conducted within enclosed zones	Yes	Yes	Yes
Mainland Italy	January 2022	Ongoing	Yes, partially and delayed	Hunting restrictions not stable; collective and driven hunting allowed during specific periods	Yes; driven hunting with a maximum of three dogs per hunter or hunting group	Yes	Yes	Implemented, but not systematically
Latvia	June 2014	Ongoing	N/A	No hunting restrictions	N/A	Yes	No	Yes
Hungary	April 2018	Ongoing	N/A	Individual hunting permitted in Restriction Zone I	Yes; detailed strategies not reported	Yes	Implemented, but not systematically	N/A
Greece	February 2020	Ongoing	N/A	Hunting permitted only in selected parts of restricted areas	N/A	Yes	N/A	N/A
Romania	July 2017	Ongoing	N/A	No hunting restrictions	N/A	Yes	Yes	Yes
Poland	February 2014	Ongoing	Yes, partially and not timely	Hunting permitted within all restricted areas	Yes; detailed strategies not reported	Yes	Implemented, but not systematically	Yes
Slovakia	July 2019	Ongoing	N/A	Hunting permitted in restricted areas; individual hunting in Zone II, collective hunting in Zone I and buffer zones	N/A	Yes	Implemented, but not systematically	Yes
Estonian	September 2014	Ongoing	N/A	Reinforced hunting and financial incentives	Implemented, but not systematically	Yes	Implemented, but not systematically	Yes
Lithuania	January 2014	Ongoing	N/A	Reinforced hunting and financial incentives.	Implemented, but not systematically	Yes	Implemented, but not systematically	Yes

## Data Availability

No new data were created or analyzed in this study. Data sharing is not applicable to this article.

## Supplementary Materials

The following supporting information can be downloaded at: https://www.mdpi.com/article/10.3390/vetsci13040340/s1, Table S1: Results of PUBMED research and Selected Manuscript for the scope.
